# Bacterial surface lipoproteins mediate epithelial microinvasion by *Streptococcus pneumoniae*

**DOI:** 10.1128/iai.00447-23

**Published:** 2024-04-17

**Authors:** Jia Mun Chan, Elisa Ramos-Sevillano, Modupeh Betts, Holly U. Wilson, Caroline M. Weight, Ambrine Houhou-Ousalah, Gabriele Pollara, Jeremy S. Brown, Robert S. Heyderman

**Affiliations:** 1 Research Department of Infection, Division of Infection and Immunity, University College London, London, United Kingdom; 2 UCL Respiratory, Division of Medicine, University College London, London, United Kingdom; University of Illinois Chicago, Chicago, Illinois, USA

**Keywords:** *Streptococcus pneumoniae*, host-microbe interactions, microinvasion, bacterial lipoproteins, epithelium

## Abstract

*Streptococcus pneumoniae*, a common colonizer of the upper respiratory tract, invades nasopharyngeal epithelial cells without causing disease in healthy participants of controlled human infection studies. We hypothesized that surface expression of pneumococcal lipoproteins, recognized by the innate immune receptor TLR2, mediates epithelial microinvasion. Mutation of *lgt* in serotype 4 (TIGR4) and serotype 6B (BHN418) pneumococcal strains abolishes the ability of the mutants to activate TLR2 signaling. Loss of *lgt* also led to the concomitant decrease in interferon signaling triggered by the bacterium. However, only BHN418 *lgt::cm* but not TIGR4 *lgt::cm* was significantly attenuated in epithelial adherence and microinvasion compared to their respective wild-type strains. To test the hypothesis that differential lipoprotein repertoires in TIGR4 and BHN418 lead to the intraspecies variation in epithelial microinvasion, we employed a motif-based genome analysis and identified an additional 525 a.a. lipoprotein (pneumococcal accessory lipoprotein A; *palA*) encoded by BHN418 that is absent in TIGR4. The gene encoding *palA* sits within a putative genetic island present in ~10% of global pneumococcal isolates. While *palA* was enriched in the carriage and otitis media pneumococcal strains, neither mutation nor overexpression of the gene encoding this lipoprotein significantly changed microinvasion patterns. In conclusion, mutation of *lgt* attenuates epithelial inflammatory responses during pneumococcal-epithelial interactions, with intraspecies variation in the effect on microinvasion. Differential lipoprotein repertoires encoded by the different strains do not explain these differences in microinvasion. Rather, we postulate that post-translational modifications of lipoproteins may account for the differences in microinvasion.

**IMPORTANCE:**

*Streptococcus pneumoniae* (pneumococcus) is an important mucosal pathogen, estimated to cause over 500,000 deaths annually. Nasopharyngeal colonization is considered a necessary prerequisite for disease, yet many people are transiently and asymptomatically colonized by pneumococci without becoming unwell. It is therefore important to better understand how the colonization process is controlled at the epithelial surface. Controlled human infection studies revealed the presence of pneumococci within the epithelium of healthy volunteers (microinvasion). In this study, we focused on the regulation of epithelial microinvasion by pneumococcal lipoproteins. We found that pneumococcal lipoproteins induce epithelial inflammation but that differing lipoprotein repertoires do not significantly impact the magnitude of microinvasion. Targeting mucosal innate immunity and epithelial microinvasion alongside the induction of an adaptive immune response may be effective in preventing pneumococcal colonization and disease.

## INTRODUCTION

*Streptococcus pneumoniae* (pneumococcus) is a versatile pathobiont capable of asymptomatically colonizing the nasopharynx, causing localized infections of the middle ear, respiratory tract, and lungs, and causing disseminated invasive disease (e.g., bacteremic pneumonia and meningitis) with high mortality rates (1). *S. pneumoniae* has long been considered an extracellular pathogen despite the demonstration of bacterial invasion *in vitro* using epithelial and endothelial cell lines (1). However, controlled human infection with a serotype 6B strain revealed that the pneumococcus invades the nasopharyngeal epithelium of healthy carriers, stimulating epithelial inflammation without causing overt symptoms or disease (2–4). We have termed this phenomenon microinvasion, which is distinct from the invasion of deeper tissues or dissemination systemically which characterizes disease (2). Inflammation triggered by the epithelium-associated and intracellular bacteria, which peaks 9 days post inoculation, may be important for clearance and onward transmission (2).

In this study, we explored the hypothesis that surface expression of pneumococcal lipoproteins mediates epithelial microinvasion. Pneumococcal lipoproteins are post-translationally lipidated surface proteins, many of which function as metabolite transporters (5, 6). *S. pneumoniae* lipoproteins have also been shown to be major TLR2 ligands in macrophages, required for a Th17 response and for many of the dominant macrophage gene transcriptional responses, such as induction of IRAK-4-dependent protective cytokines (7–9). *S. pneumoniae* encodes over 30 lipoproteins, including the bifunctional adhesin/manganese transporter PsaA and the peptidoglycan hydrolase DacB (5, 10–12). Blocking lipidation by mutating the prolipoprotein diacylglyceryl transferase encoding gene *lgt* de-anchors lipoproteins from the cell surface, resulting in the release of immature preprolipoproteins into the extracellular milieu and abolishing the ability of the bacteria to activate TLR2 signaling (8, 9, 13). Mutating *lgt* also attenuates pneumococcal virulence and shortens colonization duration in murine models (8, 14).

To explore whether heterogeneity in surface-expression of pneumococcal lipoproteins also explains the differences in microinvasion seen between strains, we blocked lipoprotein lipidation by inactivation of *lgt* in two well-characterized strains: a highly invasive strain (TIGR4, serotype 4) and a less invasive strain (BHN418, serotype 6B) which was used in the controlled human challenge experiments (15, 16). It is important to note that pneumococcal strains from both serotypes can asymptomatically colonize as well as cause invasive disease in susceptible hosts, albeit to different extents (17). While attenuation of inflammatory responses was seen with both serotype 6B and serotype 4 *lgt* mutants, we observed intraspecies differences in the contribution of lipoproteins to microinvasion, with greater effects of lipoproteins with the less invasive 6B strain. Genomic analysis revealed the presence of a previously uncharacterized lipoprotein encoded within a genetic island found in BHN418 and approximately 10% of pneumococcal strains, but not in TIGR4. We designate this protein pneumocccal accessory lipoprotein A, or PalA, and investigated its role in mediating intraspecies differences in microinvasion.

## RESULTS

### Pneumococcal *lgt* mutants induce lower levels of TLR2 and interferon signaling compared to wild-type strains

**Fig 1:**
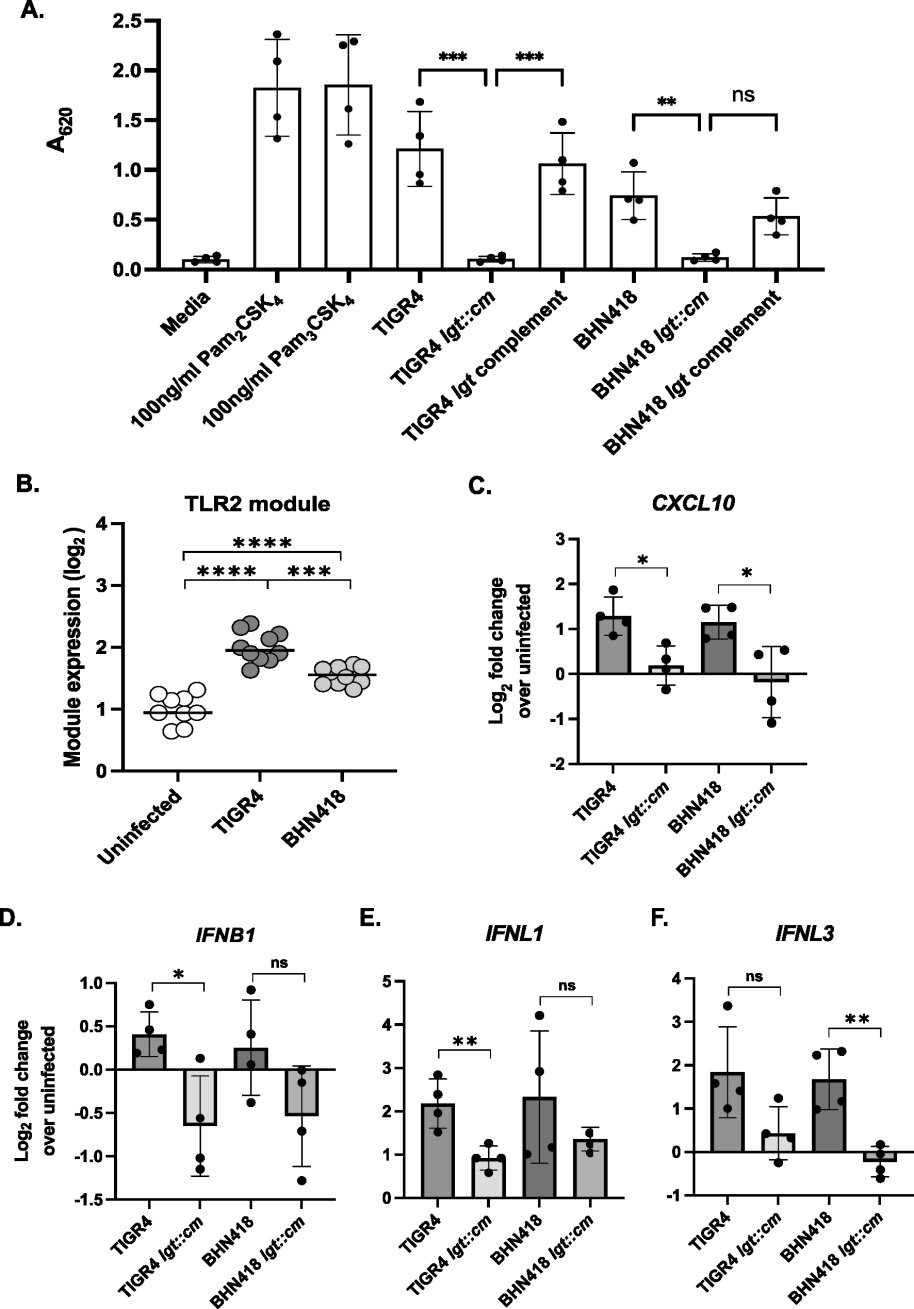
Pneumococcal *lgt* mutants were less inflammatory compared to WT strains. (**A**) SEAP reporter readout from HEK-Blue hTLR2 reporter cells treated with pneumococcal strains at MOI 10 for 16 hours. (**B**) Expression of a transcriptional module reflective of TLR2-mediated activity in Detroit 562 cells infected with TIGR4 and BHN418 for 3 hours. (**C–F**) Transcript levels of (**C**) *CXCL10*, (**D**) *IFNB1*, (**E**) *IFNL1*, and (**F**) *IFNL3*, quantified via qPCR using total RNA extracted from Detroit 562 cells after 6 hours of infection with pneumococcal strains. Statistical significance was determined using multiple comparison test with Bonferroni’s correction (**A**), Mann-Whitney test (**B**), or Student’s *t* test assuming equal variance (**C–F**). * indicates *P* < 0.05, ** indicates *P* < 0.01, *** indicates *P* < 0.001, and *** indicates *P* < 0.0001.

In line with previous reports, mutation of *lgt* in both TIGR4 and BHN418 completely abolished the ability of these strains to trigger TLR2 signaling in HEK-Blue hTLR2 reporter cells, while genetic complementation of *lgt* at a chromosomal ectopic site restored wild-type (WT)-like ability to stimulate the TLR2 pathway (Fig. 1A) (9). Although macrophages respond to pneumococcal infections by activating TLR2 signaling pathways, it is unknown if nasopharyngeal epithelial cells respond in the same way (8, 9, 18). Using a transcriptional module reflective of TLR2 signaling and previously published transcriptomic data sets (2, 19), we found evidence of elevated TLR2-mediated transcriptional activity in Detroit 562 nasopharyngeal epithelial cells infected with TIGR4 and BHN418 (Fig. 1B; Fig. S1).

TLR2 activation is necessary for the full induction of TLR4 by the *S. pneumoniae* virulence factor pneumolysin (20, 21). Transcriptomic analyses of human nasal biopsy samples from controlled pneumococcal challenge experiments and nasopharyngeal cell lines infected with *S. pneumoniae* also showed upregulation of interferon signaling (6, 7). We, therefore, hypothesize that TLR2 activation potentiates interferon signaling in epithelial cells triggered by *S. pneumoniae* infection. Using qPCR, we observed that Detroit 562 cells infected with TIGR4 *lgt::cm* have reduced expression of *CXCL10*, *IFNB1*, and *IFNL1* compared to cells infected with WT TIGR4 (Fig. 1C through E), while cells infected with BHN418 *lgt::cm* have reduced expression of *CXCL10* and *IFNL3* compared to those infected with WT BHN418 (Fig. 1C and F). Our results suggest that lipoprotein-mediated TLR2 activation augments the epithelial interferon response during pneumococcal microinvasion.

### Mutation of *lgt* attenuates epithelial microinvasion by *S. pneumoniae* serotype 6B but not by serotype 4

**Fig 2:**
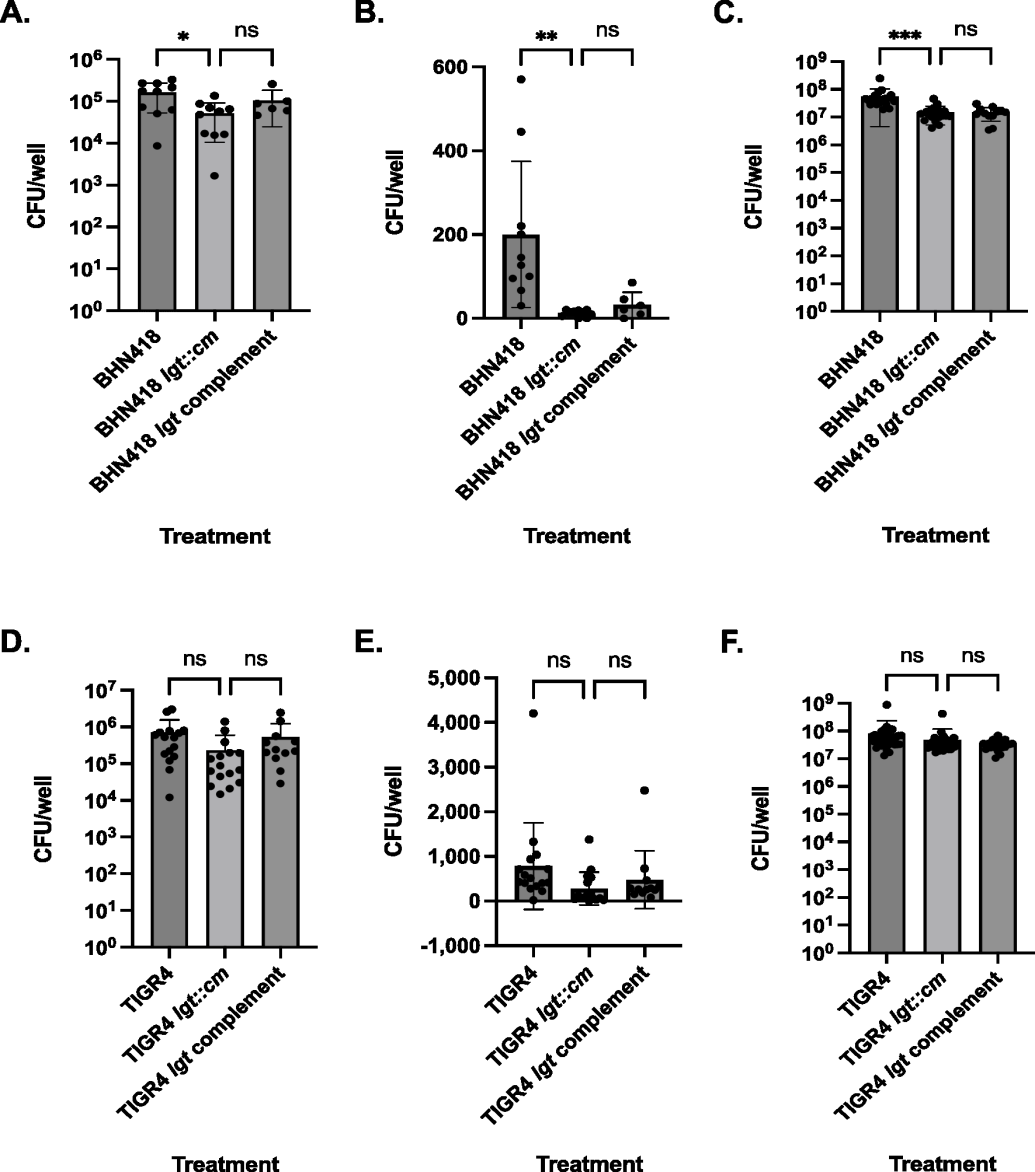
Mutation of *lgt* impaired epithelial microinvasion by *S. pneumoniae*, with greater defects for BHN418. (**A–F**) NPE microinvasion by WT and *lgt* mutants. Graphs show CFU numbers for BHN418-derived (**A–C**) and TIGR4-derived strains (**D–F**) associated with (**A and D**), internalized into (**B and E**), or growing in proximity with Detroit 562 NPE cells (C and F) 3 hours post infection. * indicates *P* < 0.05, ** indicates *P* < 0.01, and *** indicates *P* < 0.001.

To determine if mutation of *lgt* and loss of TLR2 signaling impact on pneumococcal microinvasion, we infected confluent Detroit 562 nasopharyngeal cells (NPE) with serotype 6B (BHN418) and serotype 4 strains (TIGR4) for 3 hours (3 hpi), measuring the number of cell-associated, intracellular, and planktonic bacteria in the cell culture supernatant. Mutation of *lgt* significantly attenuated the ability of BHN418 but not TIGR4 to associate with and be internalized into Detroit 562 cells (Fig. 2A, B, D, and E). In concordance with prior reports, serotype 4 strains were more invasive compared to serotype 6B strains, with ~5 times more intracellular WT TIGR4 recovered compared to WT BHN418 (Fig. 2B and D) (15, 17). The *lgt* mutation also significantly reduced the number of planktonic BHN418 but not TIGR4 (Fig. 2F).

Genetic complementation of *lgt* in the BHN418 *lgt::cm* mutant did not fully restore microinvasion of NPE cells to WT-like levels, despite complementation in the HEK-Blue hTLR2 reporter assay (Fig. 1A and 2A through F). We double-checked the strain genotype using Illumina sequencing and confirmed *lgt* transcription (or lack thereof) in the TIGR4 and BHN418 strains using semi-quantitative PCR (Table S1; Fig. S2A and B). Leaky expression of the *P*_IPTG_ promoter in the absence of an inducer is sufficient to result in *lgt* expression (Fig. S2A and B). In agreement with prior studies, immunoblotting revealed substantially reduced retention but not complete loss of lipoproteins such as PiuA in whole-cell lysates in the *lgt::cm* mutant compared to wild-type and complementation strains (Fig. S3A and B) (13, 14). We conclude that the lack of complementation for the microinvasion phenotype is not due to failure in genetic complementation.

**Fig 3:**
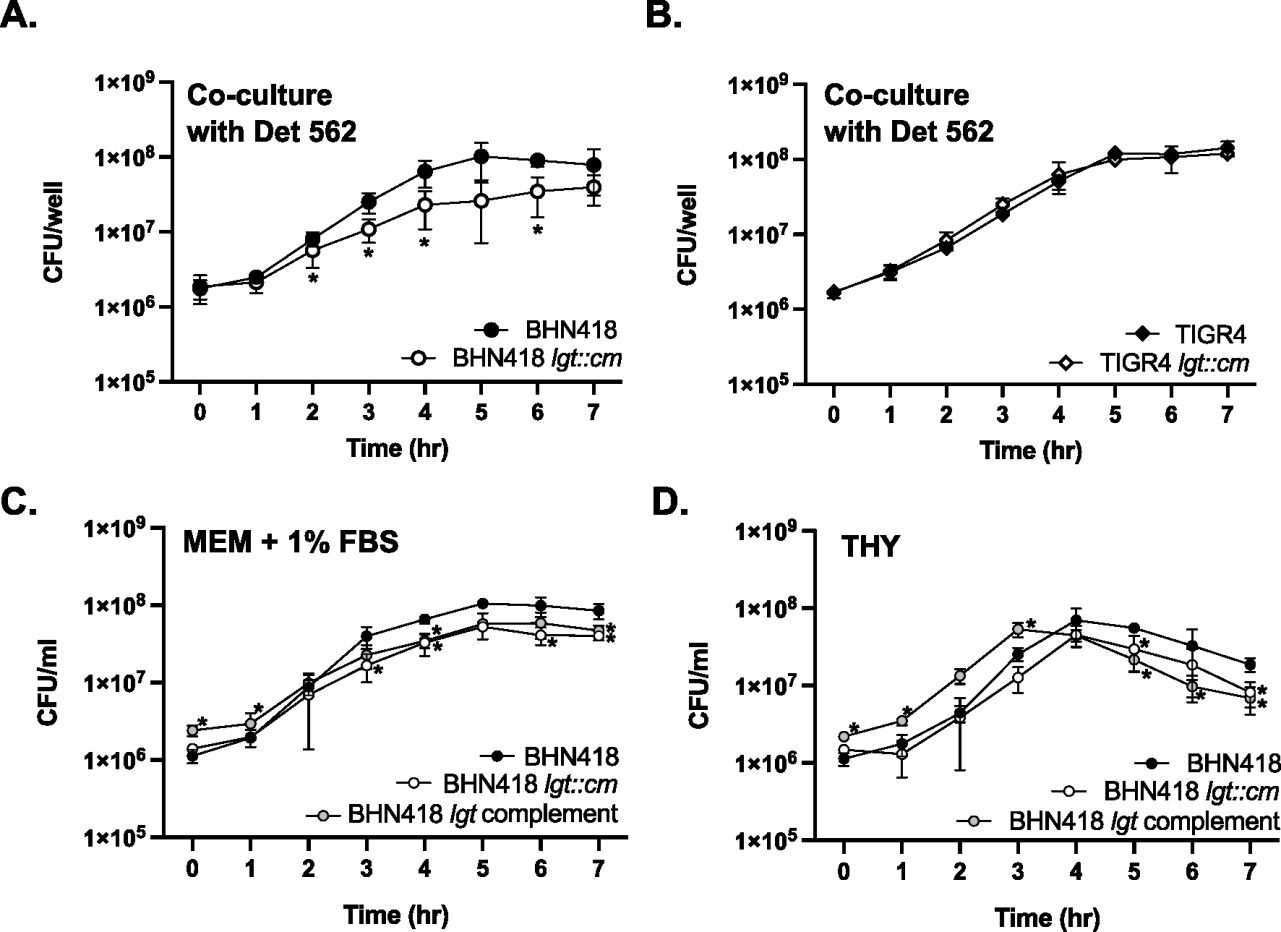
Growth of BHN418 *lgt::cm* compared to WT. (**A and B**) Growth of BHN418-derived (**A**) and TIGR4-derived (**B**) strains in infection medium (MEM + 1% FBS) in the presence of Detroit 562 NPE cells. (**C and D**) Growth of BHN418-derived strains in infection medium (**C**) and in the rich growth medium THY (**D**) in the absence of NPE cells. * indicates *P* < 0.05 by Student’s *t* test.

Mutation of *lgt* has been associated with growth defects in cation-limiting conditions, human blood, and mouse bronchoalveolar lavage fluid (14). Fewer planktonic BHN418 *lgt* mutant bacteria were also recovered from our NPE infection experiments (Fig. 2C). Time course sampling of planktonic pneumococci grown with Detroit 562 cells revealed a minor growth defect for the BHN418 *lgt* mutant starting at 3 hpi but not for the TIGR4 *lgt* mutant (Fig. 3A and B). To determine if the growth defect was dependent on the presence of NPE cells, time course sampling of planktonic BHN418 and its *lgt* mutant grown in infection medium and rich THY medium were performed. The growth defect was replicated in a cell-free medium and is therefore not dependent on the presence of NPE cells (Fig. 3C and D).

Our results indicate that inactivation of Lgt and, therefore, the lipoprotein processing pathway had greater consequences for BHN418 compared to TIGR4, except in their ability to trigger epithelial inflammation. These observations suggest that activation of the TLR2 pathway during pneumococcal-epithelial interactions is not dependent on the number of cell-associated or intracellular pneumococci. Additionally, within the timeframe of our assays, TLR2 signaling neither promotes nor inhibits epithelial microinvasion by *S. pneumoniae*.

### BHN418 encodes a novel lipoprotein absent in TIGR4

One potential explanation for the intraspecies differences in microinvasion upon *lgt* mutation is the presence of one or more lipoproteins in BHN418 which are absent in TIGR4. This lipoprotein may play a role as an adhesin and/or be important for nutrient transport and growth during infections. To address this hypothesis, we used a motif-based sequence toolkit, the MEME Suite, to compare the lipoprotein repertoires of BHN418 and TIGR4 (22). We identified 43 open-reading frames (ORFs) in TIGR4 and 44 ORFs in BHN418 with gene products that fit the criteria for a lipoprotein (detailed in Materials and methods; Table 1). Of these putative lipoprotein ORFs, only one is present in BHN418 but not in TIGR4. There are no lipoprotein ORFs present in TIGR4 that are not also present in BHN418.

**TABLE 1:** Lipoproteins encoded by TIGR4 and BHN418, identified bioinformatically using MEME suite

BHN418 locus tag	TIGR4 locus tag	Gene name	Description of gene product	Reference
RSS80_07140	SP_1500	*aatB*	Amino acid transporter	(23)
RSS80_10715	SP_2169	*adcA*	Adhesin competence protein A; zinc transporter	(24)
RSS80_04890	SP_1002	*adcAII*	Adhesin competence protein AII; zinc transporter	(25)
RSS80_01875	SP_0366	*aliA*	AmiA-like protein A; oligopeptide transporter	(26)
RSS80_07270	SP_1527	*aliB*	AmiA-like protein B; oligopeptide transporter	(26)
RSS80_09070	SP_1891	*amiA*	Aminopterin resistance locus protein A; oligopeptide transporter	(27)
RSS80_03115	SP_0629	*dacB*	L,D-carboxypeptidase	(10)
RSS80_03240	SP_0659	*etrx1*	Extracellular thioredoxin-like protein 1; thiol-disulfide oxidoreductase	(28)
RSS80_04875	SP_1000	*etrx2*	Extracellular thioredoxin-like protein 2; thiol-disulfide oxidoreductase	(29)
RSS80_06715	SP_1394	*glnH*	GlnH glutamine/polar amino acid ABC transporter substrate-binding protein	(30)
RSS80_00785	SP_0148	*gshT*	Glutathione transporter	(31)
RSS80_03685	SP_0749	*livJ*	Branched chain amino-acid transporter	(32)
RSS80_10385	SP_2108	*malX*	Maltosaccharide transporter	(33)
RSS80_00790	SP_0149	*metQ*	Methionine-binding lipoprotein Q	(34)
RSS80_05810	SP_1175	*phtA*	Pneumococcal histidine triad protein A	(35)
RSS80_05040	SP_1032	*piaA*	Pneumococcal iron acquisition protein A	(36)
RSS80_01305	SP_0243	*pitA*	Pneumococcal iron transporter protein A	(37)
RSS80_08955	SP_1872	*piuA*	Pneumococcal iron uptake protein A	(36)
RSS80_04120	SP_0845	*pnrA*	Nucleoside transporter	(38, 39)
RSS80_04795	SP_0981	*ppmA*	Putative proteinase maturation protein A; peptidyl-prolyl *cis*–*trans* isomerase	(40)
RSS80_07850	SP_1650	*psaA*	Pneumococcal surface adhesin A; manganese and zinc transporter	(11, 12)
RSS80_10265	SP_2084	*pstS*	Phosphate transport substrate-binding protein	(41)
RSS80_09095	SP_1897	*rafE*	Raffinose transporter	(42)
RSS80_03790	SP_0771	*slrA*	Streptococcal lipoprotein rotamase A;cyclophilin-type peptidyl-prolyl *cis*–*trans* isomerase	(43)
RSS80_04895^*c*^	SP_1003	*phtB*	Pneumococcal histidine triad protein B	(35)
RSS80_04895^*c*^	SP_1174	*phtD*	Pneumococcal histidine triad protein D	(35)
RSS80_06745	SP_1400	*pstS2*	phosphate binding protein	–
RSS80_10885	SP_2197	*–^d^*	ABC transporter binding protein	–
RSS80_00530	SP_0112	*–*	Amino acid-binding protein	–
RSS80_09960	SP_2041	–	Membrane protein insertase	–
RSS80_04185	SP_0857^*b*^	–	ABC transporter substrate-binding protein	–
RSS80_03070	SP_0620	*–*	Amino acid ABC transporter binding protein	–
RSS80_09570	SP_1975	–	Membrane protein insertase	–
RSS80_03435^*b*^	SP_0708^*b*^	–	ABC transporter substrate-binding protein (truncated)	–
**RSS80_03595**	**Not present**	**–**	**Extracellular solute binding protein**	**–**
RSS80_04430	SP_0899	*–*	Hypothetical protein	–
RSS80_05800^*b*^	Not assigned	–	ABC transporter substrate-binding protein (truncated)	–
RSS80_08015	SP_1683	*–*	ABC transporter sugar-binding protein	–
RSS80_08055	SP_1690	*–*	ABC transporter sugar-binding protein	–
RSS80_08595	SP_1796	–	Extracellular solute binding protein	–
RSS80_08765	SP_1826	–	ABC transporter substrate-binding protein	–
RSS80_00445	SP_0092	*–*	ABC transporter substrate-binding protein	–
RSS80_01055	SP_0191	*–*	Hypothetical protein	–
RSS80_01080	SP_0198	*–*	ABC transporter substrate-binding protein	–

^*a*^ Bold: ORF present in BHN418 but not TIGR4. Not assigned: Homologous sequence present in genome but not annotated as ORF/CDS. Not present: Homologous sequence absent in genome. TIGR locus tag and gene name are based on TIGR4 genome annotation (Genbank accession number AE005672.3).

^*b*^ Annotated as pseudogene, contained premature stop codon, or interrupted by insertion sequence.

^*c*^ RSS80_04895 was the best match BLAST result for more than one TIGR4 CDS.

^*d*^ –, no known common gene name for the designated gene/locus.

**Fig 4:**
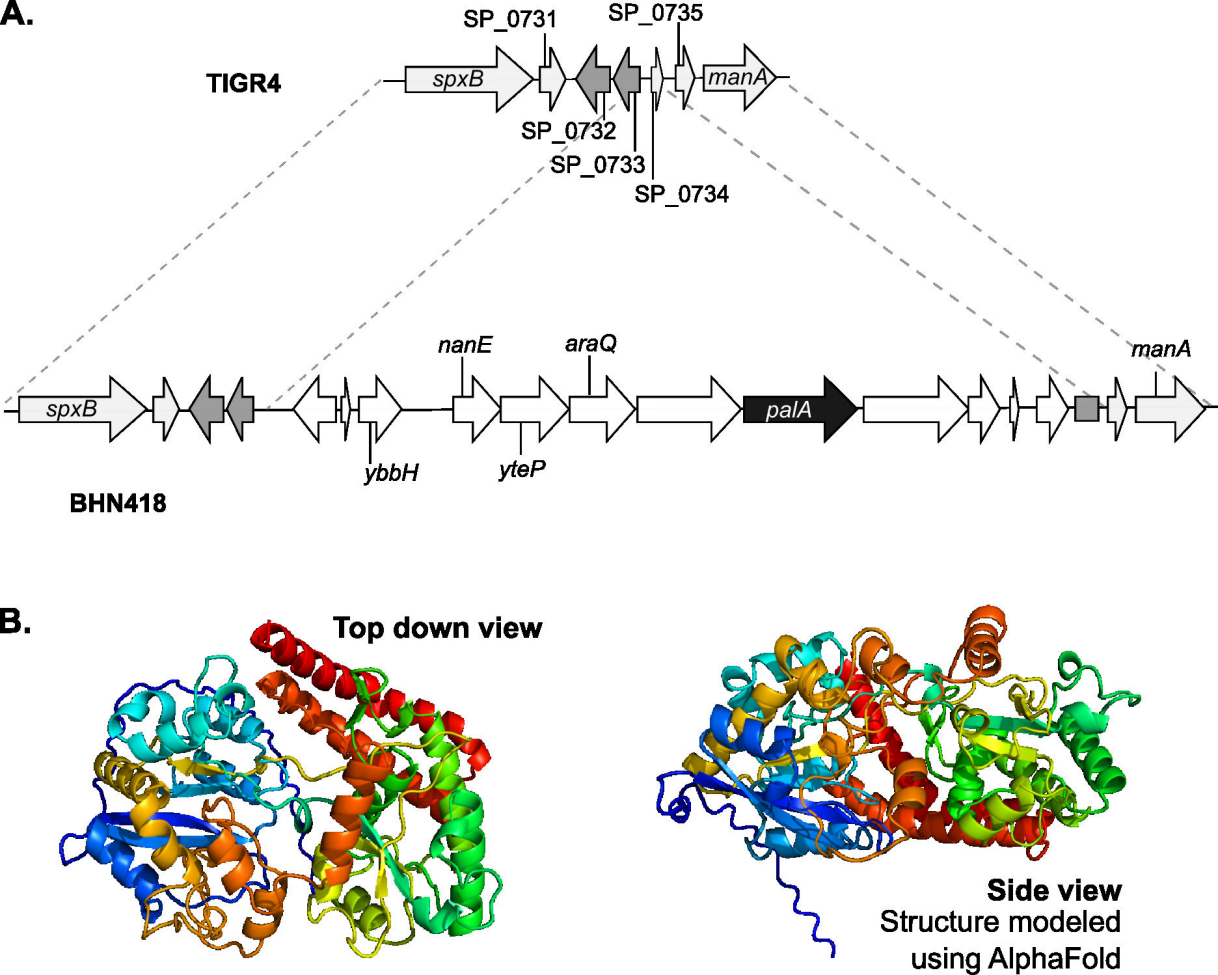
PalA is a lipoprotein present in BHN418 but not in TIGR4. (**A**) Genetic context of the *palA* gene (black arrow), which is within a multi-gene operon that is part of a putative genetic island. ORFs with homology to previously described genes are labeled with the gene name. (**B**) Predicted structure of PalA, modeled using AlphaFold2, which shows two subdomains connected by a hinge region with a possible central ligand-binding pocket. The extended stalk-like structure contains the signal peptide and lipoprotein processing sequence and is likely cleaved/absent in the mature lipoprotein.

The lipoprotein encoded by BHN418 but not TIGR4, encoded by the gene with the locus tag RSS80_03595 and which we named pneumococcal accesssory lipoprotein A (PalA), comprises of 525 amino acids with sequence and structural homology to extracellular solute binding domain proteins that deliver substrates to ABC family transporters (Fig. 4A and B). We modeled PalA’s tertiary structure using AlphaFold2 and PHYRE, revealing a Type II periplasmic binding protein fold characterized by two subdomains connected with a hinge region (Fig. 4B; Fig. S4A) (44–46). The two predicted structures aligned well with each other, barring small conformational differences in the accessibility of the potential substrate binding pocket (Fig. S4B and C). The N-terminal extension (stalk-like structure) seen in Fig. 4B is likely cleaved post-lipidation (5).

Periplasmic (extracellular) binding proteins deliver substrates to ABC transporters, which are multi subunit proteins comprising of two transmembrane permease domains and two cytoplasmic ATPase domains (47). Two genes encoding ABC transporter permease domain proteins, annotated as *yteP* and *araQ*, were found ~3.3 kb and ~2.4 kb upstream of *palA.* We were unable to locate ORF(s) encoding for the ATPase domain proteins in the 10 kb region upstream or downstream of *palA*. Taken together, PalA likely binds to and delivers substrate(s) to YteP and/or AraQ. It is uncertain if YteP and/or AraQ co-opt the ATPase domains of ABC family transporters encoded elsewhere on the genome, in a similar strategy as the raffinose utilization system, or no longer function as transporters (42, 48).

This genetic context suggests that *palA* is the fifth gene in an operon encoding for carbohydrate import and utilization genes, which includes *yteP* and *araQ* (Fig. 4A). Upstream of the operon is a gene encoding a putative transcriptional regulator, *ybbH*, which may play a role in regulating the expression of the operon. The operon and *ybbH* sit within an 11.5 kb region with ~30% GC content, flanked by repetitive insertion sequences with homology to *IS630* elements. This region was likely acquired via horizontal gene transfer as the overall mean GC content of pneumococcal strains is around 40% (49). This putative genetic island is directly downstream of *spxB*, which encodes an important pneumococcal virulence factor involved in the production of H_2_O_2_ (50). Alignment of TIGR4 whole-genome sequencing reads to the BHN418 genome revealed the absence of the entire putative island in the TIGR4 genome (Fig. S5; 49).

To determine if *palA* is predominantly present in more carriage-type serotypes, such as serotype 6B, we examined the presence of *palA* in a well-curated data set of 2,806 carriage isolates from Malawi (51). Five hundred sixty-seven of these carriage isolates (20.5%) carry *palA* in their chromosome. Mapping the analysis results onto a hierarchal clustering (Newick) tree showed that *palA* is present in specific lineages, with no clear association to capsular serotypes or sequence types (genetic relatedness, visualized as neighboring branches on a Newick tree; Fig. S6) (51). However, the presence of *palA* is enriched in certain serotypes, particularly serotype 6A (39/93, 41.9%), 6B (12/31, 38.7%), 10A (29/34, 85%), 15B (52.76, 68.4%), 16F (60/94, 63.8%), 23B (45/103, 43.7%), 35A (28/28, 100%), and 35B (60/114, 52.6%; Table S2). Additionally, the branching patterns of the phylogenetic tree for the Malawi carriage isolates support the inference that *palA* and its associated genetic island were acquired via horizontal gene transfer and expanded in specific lineages (Fig. S6).

### PalA presence is enriched in carriage and ear isolates

Maintenance of this 11.5 kb genetic island is potentially costly and suggests that the island confers some form of advantage to isolates that carry it. *S. pneumoniae* is capable of colonizing and infecting multiple body sites including the nasopharynx, lungs, blood, cerebrospinal fluid, meninges, and middle ear. We, therefore, examined 51,379 genomes in the BIGSdb database to determine if there is an association between the presence of *palA* and the isolation site of the strain (“source”) (52).

The *palA* gene presence is enriched in carriage isolates and in strains isolated from ear infections compared to strains isolated from invasive pneumococcal disease (IPD) or lower respiratory tract disease (Table 2). More than half of serotype 22F and 6A strains isolated from the ear carried *palA* and approximately 28% of all serotype 22F and 6A genomes in the database carry *palA*, in contrast to the overall *palA* prevalence rate of 9.97% (Table 2). By contrast, *palA* was detected in only 6.7% of hypervirulent serotype 1 genomes hosted on the BIGSdb database and in only 5 of 895 genomes belonging to the multidrug-resistant lineage GPSC10 on the Global Pneumococal Sequencing database (~0.55%). In the BIGSdb database, the only serotype 4 strain isolated from the ear carried *palA* in its genome. These observations suggest that *palA* and/or its putative genetic island may facilitate spread to and cause infection of the ear, although *palA*’s presence is not necessary for colonization of the ear.

**TABLE 2:** Presence of *palA* in whole-genome sequences of pneumococcal isolates on the BIGSdb database, stratified by site of isolation (“source”)

Category	Source (BIGSdb label)	Proportion (%)
Carriage	“Nasopharynx,” “pharynx,” and “sputum”	14.57 (3,114/21,369)
Otitis	“Ear swab” and “middle ear fluid”	13.72 (129/940)
Pneumonia	”Lung aspirate,” “sinus aspirate,” “bronchoalveolar lavage,” and “bronchi”	8.96 (25/279)
Invasive	“Blood,” “cerebrospinal fluid,” “joint fluid,” and “pleural fluid”	6.54 (1,837/28,075)
Eye/pus/others	“eye swab,” “pus,” and “other”	2.65 (19/716)
Overall		9.97 (5,124/51,379)

### Mutation of *palA* does not alter pneumococcal colonization or microinvasion of the epithelium

**Fig 5:**
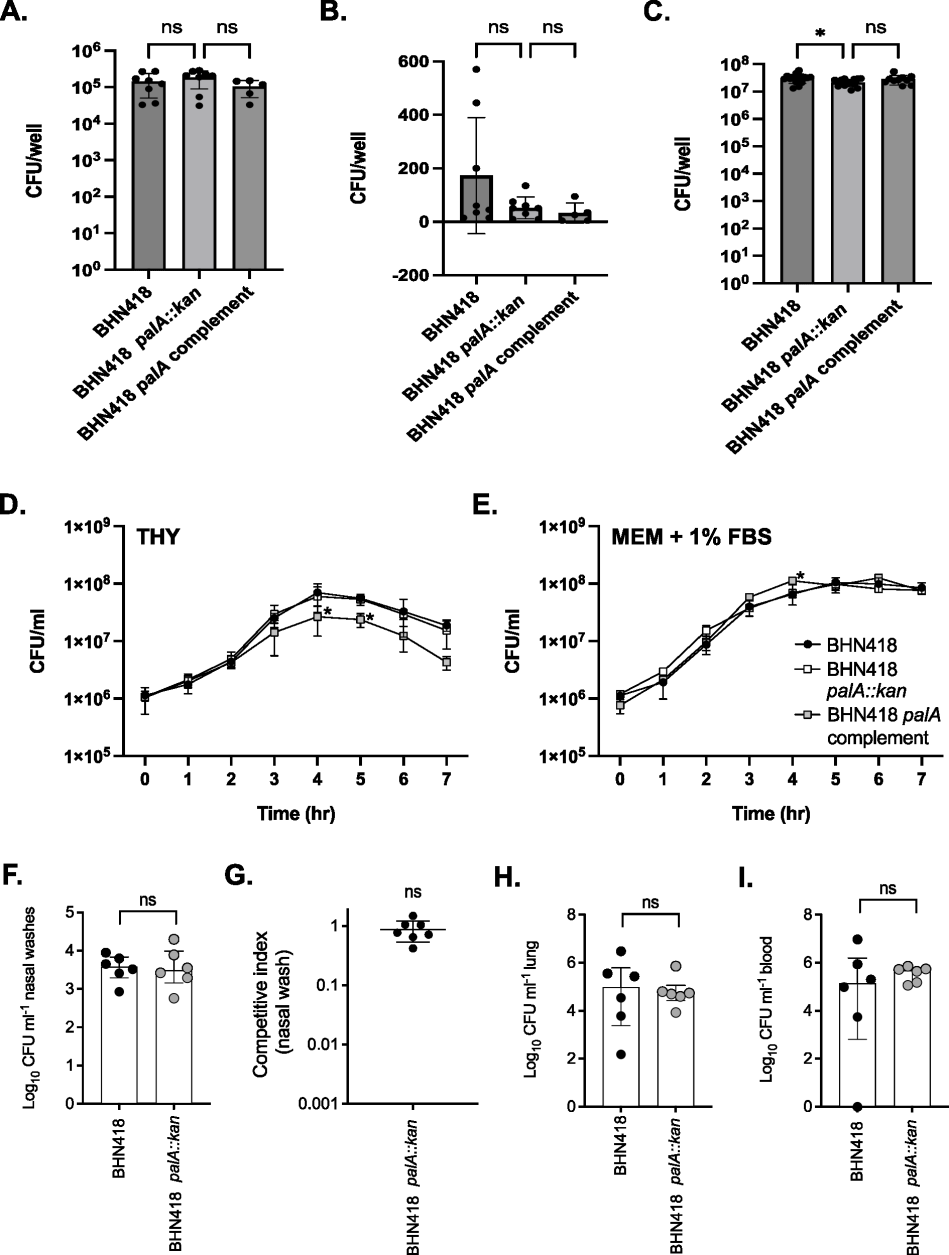
PalA was not essential for NPE microinvasion, murine colonization, and progression to disease. (**A–C**) NPE microinvasion by WT BHN418 and *palA* mutants, measured as NPE-associated bacteria (**A**), internalized bacteria (**B**), and planktonic bacteria growing in proximity with Detroit 562 NPE cells 3 hours post infection. (**D and E**) Growth of WT BHN418, the *palA* knock out, and complementation mutants in THY (**D**) and infection medium (**E**). (**F–I**) Recovery of pneumococci from mice intranasally inoculated with WT BHN418 and *palA::kan* mutant, recovered from nasal washes when inoculated singly (**F**) or competitively in a 1:1 ratio (**G**), as well as from the lungs (**H**) and bloodstream (**I**) when tested on a pneumonia model. * indicates *P* < 0.05.

To determine if PalA plays a role in epithelial microinvasion, we generated *palA* deletion and complementation mutants for testing in our NPE model. The deletion and complementation mutants were verified using Sanger sequencing, Illumina sequencing, and semi-quantitative PCR of *palA* transcript (Table S1; Fig. S2). Although there is a small reduction in the number of planktonic bacteria, the numbers of epithelial-associated and intracellular BHN418 *palA::kan* were not significantly different from that of WT BHN418 (Fig. 5A through C). Additionally, we did not observe a growth defect when BHN418 *palA::kan* was grown in THY or infection medium (Fig. 5D and E).

Mutation of *lgt* attenuates nasopharyngeal colonization density and duration in mice (14). We next asked if the presence of *palA* confers a survival advantage in a more complex and immune-replete environment such as the murine nasopharynx. Outbred CD-1 female mice were intranasally inoculated with wild-type BHN418 and the *palA* mutant either singly or in a 1:1 competitive mix. After 7 days of colonization, similar CFU numbers for WT BHN418 and the *palA::kan* were recovered from nasal washes (Fig. 5F and G). Similar CFU numbers for BHN418 and *palA::kan* were also recovered in homogenized lungs and blood 24 hours post inoculation in a murine pneumonia model (Fig. 5H and I). We conclude that the presence of *palA* does not confer a colonization advantage in the murine nasopharynx or in the progression to bacteremic pneumonia.

**Fig 6:**
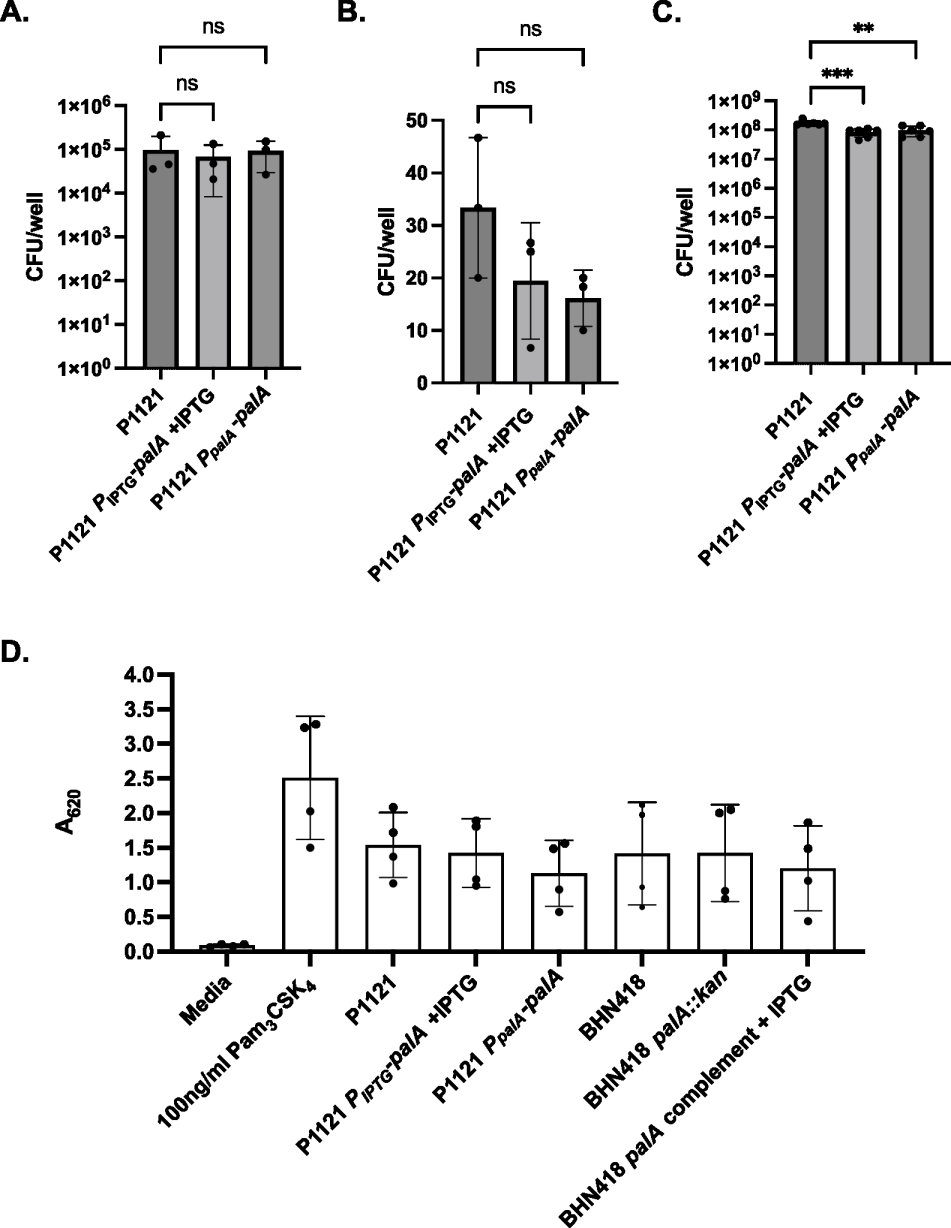
Heterologous expression of *palA* in P1121 (serotype 23F) did not increase epithelial microinvasion or TLR2 signaling. (**A–C**) NPE microinvasion by WT P1121 and *palA* expression mutants, measured as NPE-associated bacteria (**A**), internalized bacteria (**B**), and planktonic bacteria growing in proximity with Detroit 562 NPE cells 3 hours post infection (C). (**D**) SEAP reporter readout from HEK-Blue hTLR2 reporter cells treated with pneumococcal strains at MOI 10 for 16 hours.

To further probe the function of PalA, we heterologously expressed *palA* in a serotype 23F strain naturally lacking the island (P1121). Semi-quantitative PCR of *palA* transcripts demonstrated substantial *palA* expression driven by the inducible promoter (Fig. S2C). Expression of *palA* in P1121 did not increase the microinvasion potential of the resulting strains and reduced the number of planktonic bacteria in the cell culture supernatant (Fig. 6A through C). Moreover, the BHN418 *palA* knockout strains and the P1121 *palA* knock-in strains activated TLR2 signaling to similar levels as their respective wild-type strains (Fig. 6D). We, therefore, conclude that the presence of *palA* is not solely responsible for the observed strain-specific differences in Lgt-mediated epithelial microinvasion. Moreover, PalA does not contribute significantly to pneumococci’s ability to activate TLR2.

## DISCUSSION

In this study, we demonstrated that pneumococcal lipoproteins trigger inflammation during epithelial colonization at least partially via the TLR2-dependent pathway. We have previously shown that epithelial microinvasion can occur in the absence of disease, and there is heightened epithelial inflammation around the time of pneumococcal clearance in controlled human infection (2). In murine models, mutation of *lgt* reduces carriage duration and attenuates disease, associated with a concomitant reduction in inflammatory and immune responses (8, 9, 53). Although we have shown that BHN418 *lgt::cm* but not TIGR4 *lgt::cm* was significantly attenuated in epithelial adherence and microinvasion compared to their respective wild-type strains, this does not appear to be TLR2-dependent or due to differential lipoprotein repertoires encoded by the strains.

We additionally observed that the presence of *lgt* and therefore TLR2 activation heightens the epithelial interferon response elicited by pneumococcal microinvasion in the absence of immune cells. Induction of the interferon pathway upon pneumococcal challenge is thought to be dependent on sensing of intracellular pneumococci, pneumococcal DNA, or cellular DNA damage by the infected cells (54–59). In infant mice co-infected with pneumococci and influenzae, interferon signaling increases bacterial shedding while protecting against invasive disease, while TLR2 signaling limits bacterial shedding and transmission (18, 60, 61). Since TLR2 signaling augments the interferon response by human nasopharyngeal epithelial cells during mono-pneumococcal infection, it is unclear how these two pathways mediate the outcomes of pneumococcal microinvasion, colonization, or progression to disease in people. Nonetheless, our results suggest that mucosal innate immunity could be targeted alongside the induction of an adaptive immune response to prevent pneumococcal colonization, transmission, and invasive disease.

We have implicated lipoprotein expression in intraspecies differences in NPE cell microinvasion, but our genetic and mutational analysis lead us to suggest that this is not due to differences in lipoprotein repertoire but potentially due to differential post-translational lipidation kinetics. The lipoprotein PsaA functions as a bacterial adhesin that binds to host cell E-cadherin (62). In theory, we should have observed a significant reduction in the number of epithelial-associated bacteria for both the TIGR4 and BHN418 *lgt* mutants compared to wild-type due to the loss of PsaA surface presentation; however, our data did not reflect this hypothesis. Similar subtle effects have been seen, for example, when mutating the lipoprotein encoding gene *dacB* but not *lgt* alters murein sacculus composition, and when mutating *lgt* results in strain-dependent variable effects on growth in rich medium (5, 9, 14, 53, 63). The observation that genetic complementation of *lgt* in BHN418 restores the pneumococcal ability to trigger epithelial inflammation and retain PiuA and other lipoproteins in whole-cell lysate, but does not restore WT levels of microinvasion, further suggests that regulation of lipoprotein processing may be more complex than previously thought. The assumption that Lgt and other lipoprotein modification proteins are constitutively expressed and active and all 30+ lipoproteins are processed at similar rates may not be entirely correct.

We, therefore, speculate that unlike Gram-negative bacteria, for which lipoprotein processing is essential, pneumococci and other Gram-positive bacteria may compensate for the loss of Lgt by differentially regulating the expression of lipoprotein encoding genes, which in turn is regulated by external stimuli in nasopharyngeal niche (64, 65). Proteomics and immunoblotting analyses showed that the abundance of specific lipoproteins increase, decrease, or show no change when lipoprotein processing is disrupted in *S. pneumoniae*, although no clear patterns were apparent (5, 13). Additionally, we and others have observed that mutation of *lgt* does not lead to complete loss/release of preprolipoprotein from the cell surface (Fig. S3) (5). Future investigation into whether pneumococci vary *lgt* expression or Lgt activity to thrive in different tissue niches, in tandem with compensatory regulation of lipoprotein expression, may be warranted. Such a study may reveal novel mechanisms of innate immune evasion. It would also be informative to determine whether there are strain-dependent differences in regulating the generation of TLR2 agonists (mature lipoproteins) and how this may affect the invasiveness and virulence of different pneumococcal lineages.

While investigating the intraspecies variation in the role of *lgt* in microinvasion, we discovered a previously uncharacterized lipoprotein encoding gene (*palA*) and its associated genetic island. Although *palA* presence is enriched in the carriage and otitis media isolates, we have not been able to demonstrate a clear role for PalA in epithelial microinvasion or in a murine model of colonization or disease. We evaluated bacterial burden in mice 7 days post infection and thus cannot eliminate the possibility that PalA plays a role in the early phases of colonization or in persistence. PalA is predicted by sequence and structure homology to be involved in carbohydrate or sugar transport, although we have yet to identify a substrate for PalA. Raffinose metabolism has been shown to contribute to lung vs ear tropism in serotype 3 and serotype 14 strains (48). It is, therefore, possible that *palA* functions in promoting niche specialization by facilitating the uptake and metabolism of uncommon sugars, such as raffinose, found in the nasopharynx or middle ear.

In conclusion, we demonstrated a role for pneumococcal surface lipoproteins in triggering epithelial inflammation and augmenting interferon signaling in response to pneumococcal-epithelial interactions. We show that pneumococcal lipoproteins mediate microinvasion in a strain-dependent manner, which may explain the significant attenuation in carriage duration and disease with *lgt* mutants reported by others (8, 14, 53). Additionally, we have characterized a novel accessory lipoprotein likely acquired through horizontal gene transfer but rejected the hypothesis that this lipoprotein contributes to strain differences in pneumococcal epithelial microinvasion. Instead, we postulate that differential regulation of lipoprotein gene expression responding to the nasopharyngeal niche regulates this microinvasion process.

## MATERIALS AND METHODS

### Bacterial growth and maintenance

*S. pneumoniae* strains were grown on Columbia agar base with 5% defibrinated horse blood (CBA plates; EO Labs, Oxoid) statically in Todd-Hewitt broth supplemented with 0.5% yeast extract (THY; Oxoid) or in brain heart infusion broth (BHI; Oxoid) at 37°C, 5% CO_2_. Where appropriate, growth medium was supplemented with antibiotics at the following concentrations: chloramphenicol (10 µg/mL), erythromycin (0.5 µg/mL), and kanamycin (250 µg/mL). Working stocks for infections were prepared by freezing THY cultures at OD_600_ 0.3–0.4 with 10% glycerol. NEB Stable competent *Escherichia coli*-derived strains were grown in Luria-Bertani (LB) broth or LB agar (Difco) supplemented with ampicillin (200 µg/mL) where appropriate. Bacterial strains used in this paper are listed in Table 3.

**TABLE 3:** Bacterial strains and plasmids used in this study

Designation	Genotype/description	Source
Strains		
TIGR4	WT serotype 4 isolate	(49)
BHN418	WT serotype 6B isolate	(16)
P1121	WT serotype 23F isolate	(2)
ECSPN100	TIGR4 *lgt::cm*	This work
ECSPN106	TIGR4 *lgt::cm P*_IPTG_-*lgt-erm* (TIGR4 *lgt* complementation)	This work
ECSPN200	BHN418 *lgt::cm*	This work
ECSPN210	BHN418 *lgt::cm P*_IPTG_-*lgt-erm* (BHN418 *lgt* complementation)	This work
ECSPN211	BHN418 *palA::kan*	This work
ECSPN213	BHN418 *palA::kan P*_IPTG_-*palA-erm* (BHN418 *palA* complementation)	This work
ECSPN400	P1121 *P*_IPTG_-*palA-erm*	This work
ECSPN401	P1121 *P_palA_-palA-kan*	This work
Plasmids		
pASR103	Complementation construct with an IPTG inducible promoter and *erm* selectable marker	(66)
pPEPY	Complementation construct with a *kan* selectable marker	(66)
pEMcat	Minitransposon plasmid; source of *cm^R^* cassette	(67)
pABG5	Cloning plasmid; source of *kan^R^* cassette	(68)
pEC210	TIGR4 *lgt* coding region cloned into pASR103	This work
pEC211	BHN418 *lgt* coding region cloned into pASR103	This work
pEC213	BHN418 *palA* coding region cloned into pASR103	This work
pEC213	BHN418 *palA* promoter and coding region cloned into pPEPY	This work

### Bacterial genetic manipulation

*S. pneumoniae* were genetically manipulated using a competence-stimulating peptide (CSP)-mediated transformation assay (69). Briefly, pneumococci were grown in THY pH 6.8 supplemented with 1 mM CaCl_2_ and 0.02% bovine serum albumin (BSA) at 37°C, 5% CO_2_ to OD_600_ 0.01–0.03, pelleted and resuspended in 1/12 vol THY pH 8.0 supplemented with 1 mM CaCl_2_ and 0.2% BSA. A total of 400 ng CSP (Cambridge Biosciences; CSP-2 for TIGR4; 1:1 ratio of CSP-1:CSP-2 for BHN418; CSP-1 for P1121) was added to the bacterial suspension and incubated at RT for 5 min. The suspensions were then mixed with ~300 ng transforming DNA, incubated at 37°C, 5% CO_2_ for 2 hours, and plated on CBA plates supplemented with relevant antibiotics. Antibiotic-resistant transformants were screened using colony PCR and confirmed by sequencing.

Transforming DNA for generating *lgt::cm* and *palA::kan* mutants was generated using overlap-extension PCR. Complementation and expression constructs were generated by inserting the target gene into the complementation plasmid pASR103 or pPEPY (66), which allows for integration of the construct at a chromosomal ectopic site. Plasmids used are listed in Table 3, while primers are listed in Table S3.

### Cell culture

Detroit 562 (ATCC CCL-138; human pharyngeal carcinoma epithelial cells) was expanded and maintained in MEMα (minimum essential medium; Gibco 22561021) supplemented with 10% heat-inactivated fetal bovine serum (HI-FBS; LabTech FB-1001/500 or Gibco 10438–026) at 37°C, 5% CO_2_. HEK-Blue hTLR2 reporter cells (Invivogen, hkb-htlr2) were expanded and maintained in Dulbecco’s modified Eagle medium (DMEM; 4.5 g/L glucose, 2 mM glutamine, and sodium pyruvate) supplemented with 10% HI-FBS at 37°C, 5% CO_2_. Per the manufacturer’s instructions, DMEM growth medium was supplemented with 100 µg/mL normocin and/or 1× HEK-Blue Selection (Invivogen) where appropriate.

### NPE infections

Adherence-invasion infections of confluent Detroit 562 cells with *S. pneumoniae* strains were performed at multiplicity of infection (MOI) 20 (P1121/23F derived strains) or MOI 10 (all others) for 3 hours. Working bacterial stocks were thawed, centrifuged to remove the freezing medium, and resuspended in the infection medium (MEMα with 1% HI-FBS) to the appropriate colony forming unit (CFU). One milliliter bacterial suspension was added to each well containing confluent Detroit 562 cells. Plates were incubated statically at 37°C, 5% CO_2_ for 3 hours, after which 10 µL of the supernatant was removed for CFU enumeration. For adherence assays, cells were washed thrice with phosphate buffered saline (PBS), lysed with cold 1% saponin (10 min incubation at 37°C, followed by vigorous pipetting), and 10 µL cell lysate removed for CFU enumeration. For invasion assays, cells were washed thrice with PBS, incubated with 0.5 mL infection medium supplemented with 200 µg/mL gentamicin at 37°C, 5% CO_2_ for 1 hour to kill extracellular bacteria, followed by 3× PBS wash, lysis with 1% saponin and CFU enumeration. Experiments were performed at least thrice on different days (*n* ≥ 3 biological replicates) with technical duplicates. Statistical significance was determined using one-way analysis of variance (ANOVA) with Bonferroni’s multiple comparison test.

To harvest RNA for qPCR, confluent Detroit 562 cells were treated with synthetic agonists or infected with *S. pneumoniae* strains at MOI 10 for 6 hours. Briefly, working bacterial stocks were thawed, centrifuged to remove the freezing medium, and resuspended in infection medium (MEMα with 1% HI-FBS) to the appropriate CFU. Bacterial suspensions, infection medium (negative control), or infection medium supplemented with synthetic agonists [20 µg/mL Poly(I:C); TLR3 agonist, Bio-Techne] were added to each flask. Flasks were incubated statically at 37°C, 5% CO_2_ for 6 hours, after which 10 µL were removed for CFU enumeration. Detroit 562 cells were washed thrice with PBS and harvested by scraping into 300 µL RNA*later* (ThermoFisher). For each treatment condition, RNA harvesting was performed at least thrice on different days (*n* ≥ 3 biological replicates) without technical replicates.

For growth curve experiments, *S. pneumoniae* strains were seeded into 1 mL THY or 1 mL infection medium (MEMα with 1% HI-FBS, LabTech) with and without confluent Detroit 562 cells in 12-well plates at a similar CFU number as used in infection experiments. Plates were incubated at 37°C, 5% CO_2_ for 7 hours, with aliquots taken for CFU enumeration every hour. CFU growth curves were performed at least thrice on different days (*n* ≥ 3 biological replicates) without technical replicates. Statistical significance was determined using Student’s *t* test assuming equal variance.

### Quantitative and semi-quantitative PCR

RNA from epithelial cells stored in RNA*later* was extracted using RNeasy Mini kit (Qiagen) according to manufacturer instructions. Carryover DNA was removed with TURBO DNA-*free* kit (Ambion), and cDNA was generated using LunaScript RT Supermix kit (NEB). Quantitative PCR (qPCR) was performed using Luna Universal qPCR Master Mix (NEB) in technical triplicates with primers specific for *GAPDH*, *CXCL10*, *IFNB1*, *IFNL1*, and *IFNL3* (Table S3). Whenever possible, qPCR primers were designed to span exon-exon junctions. Cycling conditions are as follows: 95°C for 5 min, 40 cycles of 95°C for 15 s, and 60.5°C for 45 s, with a plate read at the end of each cycle. Data were analyzed using the 2^ΔΔCt^ method, with media-only control and *GAPDH* levels for normalization. Statistical significance was determined using Student’s *t* test assuming equal variance.

RNA from *S. pneumoniae* stored in RNA*later* was extracted using a modified TRIzol (Ambion) protocol. Briefly, pneumococcal strains were grown in BHI to OD_600_ ~ 0.5, harvested by centrifugation at 8,000 × *g* for 8 min, resuspended in 300 µL RNA*later*, and saved at −70°C. On the extraction day, the suspensions were thawed and subjected to centrifugation at 8,000 × *g* for 8 min, followed by removal of RNA*later* and resuspension of the bacterial pellet in 1 mL TRIzol reagent. The whole 1 mL suspension was transferred to pre-chilled VK01 Precellys lysing tubes containing glass beads (Stretton Scientific) and subjected to lysis by bead beating (Precellys Evolution; 6,200 RPM, 4 × 45 s cycles with 20 s rest in between cycle). The TRIzol lysates were then centrifuged at 5,000 × *g* for 10 min at 4°C, and ~800 µL supernatant was transferred to a new, pre-chilled centrifuge tube. RNA extraction and cDNA generation were performed as described above. Semi-quantitative PCR was performed using OneTaq Quick-Load Master Mix (NEB) using primers against *lgt*, *palA*, and 16S rRNA with cycling conditions: 95°C for 5 min, 40 cycles of 95°C for 15 s, and 60.5°C for 45 s. Amplification products were analyzed using DNA gel electrophoresis.

### Immunoblotting

Five milliliter cultures of pneumococcal strains grown in BHI to OD ~ 0.5 were harvested by centrifugation at 3,900 × *g* for 15 min, resuspended in 1 mL 1× PBS with 0.1% NP-40, and transferred into pre-chilled VK01 Precellys lysing tubes containing glass beads (Stretton Scientific). Pneumococcal suspensions were lysed by bead beating (Precellys Evolution; 6,200 RPM, 4 × 45 s cycles with 20 s rest in between cycle), followed by centrifugation at 5,000 × *g* for 10 min at 4°C to pellet debris. Approximately 800 µL supernatant (whole-cell lysates) was transferred to a fresh centrifuge tube and saved at −70°C until further use. Protein concentration was determined using Bradford reagent (Thermo Scientific) per the manufacturer’s instructions and used to normalize the amount of whole-cell lysate used in immunoblotting. Lysates were mixed with loading buffer, incubated at 70°C for 10 min, and chilled on ice prior to gel loading.

Approximately 3.5 µg and 2 µg whole-cell lysate were loaded on NuPage 4%–12% Bis-Tris protein gels (Invitrogen) and subjected to gel electrophoresis and transferred onto nitrocellulose membranes. Membranes were blocked in 1× Tris-buffered saline (TBS) pH 7.4 with 0.05% Tween-20 and 5% skim milk (hereafter blocking buffer) at RT for 1 hour, washed thrice in 1× TBS pH 7.4 with 0.05% Tween-20 (wash buffer; 5 min incubation at RT per wash), and probed with antisera from mice inoculated with polysaccharide conjugated-PiuA (1:1,000) or human intravenous immunoglobulin (1:1,000) (Vigam Liquid) in blocking buffer overnight at 4°C (70). Membranes were washed thrice, incubated with IRDye 800W-conjugated polyclonal goat α-mouse or goat α-human antibody in blocking buffer (1:10,000; Abcam) at RT for 1 hour, washed thrice, and imaged using LiCor Odyssey CLx. Equal loading was checked using PonceauS staining.

### HEK-Blue hTLR2 reporter assay

HEK-Blue hTLR2 secreted alkaline phosphatase (SEAP) reporter assays were performed according to manufacturer instructions (Invivogen, hkb-htlr2). Briefly, HEK-Blue hTLR2 cells, *S. pneumoniae*, and control reagents were resuspended or diluted in pre-warmed HEK-Blue Detection medium (Invivogen). 5 × 10^4^ HEK-Blue hTLR2 cells were mixed with 5 × 10^5^ CFU *S. pneumoniae* (MOI 10) and incubated for 16 hours at 37°C, 5% CO_2_. SEAP activity was then measured spectroscopically at A_620_. One hundred nanograms per milliliter of Pam_2_CSK_4_ and Pam_3_CSK_4_ (TLR2 agonist, Bio-Techne) was used as positive controls, while a bacterium-free medium was used as a negative control. Experiments were performed at least thrice on different days (*n* ≥ 3 biological replicates) with technical triplicates. Statistical significance was determined using one-way ANOVA with Bonferroni’s multiple comparison test.

### Lipoprotein prediction using MEME suite

Amino acid sequences of 39 published D39 lipoproteins were used with the motif discovery tool MEME to identify pneumococcal lipoprotein motif(s) (5, 13, 22). The top two MEME results were combined to obtain motif: L[LA][AS][AL]LXL[AV]A**C**[SG][NQS], a modified extension of the minimal lipobox motif LAG**C** (5).

The obtained motif was used with the motif scanning tool FIMO to identify lipoproteins in the genomes of *S. pneumoniae* TIGR4, BHN418, and D39, with the latter used for quality control (71). The match *P*-value was set to 0.001. FIMO results were further filtered with the following criteria: (i) presence of the lipidated cysteine residue in the motif, (ii) presence of motif in the first 70 a.a. of the sequence, and iii) positive prediction as lipoprotein by SignalP-6.0 (72).

### Genomic analysis

The presence of *palA* and its associated genetic island was determined using Local-BLAST (BLASTN and TBLASTN) for the Malawian carriage data set (*n* = 51,379) and serotype 23F strain P1121 (73). The built-in BLAST tool on pubmlst.org was used for the analysis of the BIGSdb data set (52). BLASTN and TBLASTN tools on the NCBI database were used to identify *palA* and PalA homolog in non-pneumococcal species (73, 74). BLAST results were exported in csv format and further analyzed using R (v3.6.0) in RStudio (http://www.rstudio.com/). The presence/absence of *palA* was annotated onto a Newick tree showing the phylogeny of the Malawian carriage strains by metabolic type and visualized using iTOL (51, 75). Potential gene functions were inferred through the results of BLASTP and NCBI Conserved Domain Database searches (73, 74, 76).

The BHN418 genome assembly was generated by combining long-read sequencing (PacBio) and short-read sequencing (Illumina) methods which resulted in a single contiguous chromosome of BHN418 of length 2,107,426 bp. *De novo* assembly was performed using the Unicycler v0.4.8 pipeline in bold mode, quality assessed using QUAST v5.1.0rc1, and annotated using Bakta v1.8.2 as described previously (77–80). TIGR4 sequencing reads were aligned to the BHN418 genome using Samtools v1.14 and visualized using IGV v2.16.1 (49, 74).

Genomes of TIGR4 and/or BHN418 *lgt::cm*, *palA::kan*, and respective complementation strains were assembled *de novo* using SPAdes 3.15.5 with standard parameters and subjected to Local-BLAST (TBLASTN) to determine the presence or absence of *lgt*, *palA*, *cm*, and *kan* in the respective genomes (74, 81).

### TLR2 transcriptional module analysis

TLR2-mediated transcriptional activity in Detroit 562 cells infected with TIGR4 and BHN418 for 3 hours was determined using published RNAseq data (2). We generated a transcriptional module reflective of TLR2 activity derived from genes overexpressed in fibroblasts stimulated with TLR2 agonists Pam_2_CSK_4_ and/or FSL-1 for 6 hours relative to unstimulated controls [>1.5-fold; paired *t* test with α of *P* < 0.05 without multiple testing correction; Gene Expression Omnibus (GEO) data set GSE92466; Fig. S1A] (19). Module expression was determined by calculating the geometric mean expression of all constituent genes found in the analyzed RNAseq data set. Performance was validated using data derived from Acute Myeloid Leukemia cells (GEO data sets GSE92744) and CD14+ monocytes stimulated with Pam_3_CSK_4_ (GEO data set GSE78699; Fig. S1B and C) (82, 83).

### Murine experiments

Outbred female CD1 mice (Charles River Laboratories) were inoculated intranasally under anesthetic (isoflurane) with 1 × 10^7^ CFU bacteria (*n* = 6 for single inoculation colonization and pneumonia model, *n* = 7 for competition experiment). For colonization experiments, nasal washes were performed 7 days post infection using 1 mL PBS. For pneumonia model, mice were sacrificed 24 hpi, and bacteria were recovered from the blood and homogenized lungs. CFU numbers were enumerated using CBA supplemented with 4 µg/mL gentamicin, with an additional 250 µg/mL kanamycin where appropriate. All animal procedures were approved by the local ethical review process and conducted in accordance with the relevant UK Home Office-approved project license (PPL70/6510). Mice were housed for at least 1 week under standard conditions before use. Randomization or blinding was not performed for these experiments. Statistical significance was determined using Mann-Whitney test.

## Data Availability

BHN418, ECSPN100, ECSPN106, ECSPN200, ECSPN210, ECSPN211 and ECSPN213 genomes were deposited to NCBI with BioProject accession number PRJNA1022026 (BHN418) and PRJNA1087740 (everything else). TIGR4 sequencing reads were downloaded from the NCBI Sequence Read Archive (accession SRX6259281), while P1121 reads were downloaded from the EMBL-EBI database (accession ERS1072059) (85, 86). D39 and TIGR4 whole genome assemblies were downloaded from the NCBI GenBank database (accession numbers CP000410.2 and AE005672.3, respectively) (87). All other genomic sequences used were hosted on the PubMLST Pneumococcal Genome Library (https://pubmlst.org/organisms/streptococcus-pneumoniae/pgl) or the Global Pneumococcal Sequencing project database (https://www.pneumogen.net/gps/) (52, 75). RNAseq data used in the TLR2 transcriptional module expression analysis were obtained from the ArrayExpress database (accession E-MTAB-7841) (6).

## SUPPLEMENTAL MATERIAL

Supplemental material iai.00447-23-s0001.pdf Figures S1 to S6; Tables S1 to S3.

ASM does not own the copyrights to Supplemental Material that may be linked to, or accessed through, an article. The authors have granted ASM a non-exclusive, world-wide license to publish the Supplemental Material files. Please contact the corresponding author directly for reuse.
