# Supplementary material for: Bacterial surface lipoproteins mediate epithelial microinvasion by Streptococcus pneumoniae
Source: Infect Immun. 2024 Apr 17;92(5):e00447-23. doi: 10.1128/iai.00447-23 (PMC11075461; doi:10.1128/iai.00447-23)
Supplement: Supplemental material — Figures S1 to S6; Tables S1 to S3. [file iai.00447-23-s0001.pdf]

Supplementary materials for:

Bacterial surface lipoproteins mediate epithelial microinvasion by *Streptococcus pneumoniae*

Jia Mun Chan[a#], Elisa Ramos-Sevillano[b], Modupeh Betts[a*], Holly U. Wilson[a], Caroline M. Weight[a**], Ambrine Houhou-Ousalah[a], Gabriele Pollara[a], Jeremy S. Brown[b], Robert S. Heyderman[a#]

[a] Research Department of Infection, Division of Infection and Immunity, University College London, London, UK.
[b] Department of Respiratory Medicine, Centre for Inflammation and Tissue Repair, University College London, London, United Kingdom

A.

Genes in TLR2 module
MIR21
AQP9
NFKBIZ
CCL7
GBP1
IRAK2
METTL7A
PLA2G4A
EDNRA
TMEM140
IFIH1
PTGES
IER3
OAS3
SLC39A14
CXCL5
HERC6
NFKBIA
BTN3A3
SOD2
IL8
CXCL1
CXCL6
MIR302C
TNFAIP2
MAP3K8
GBP2
RELB
LRRN3
C8orf4
CCL2
LOC100134000
DTX3L

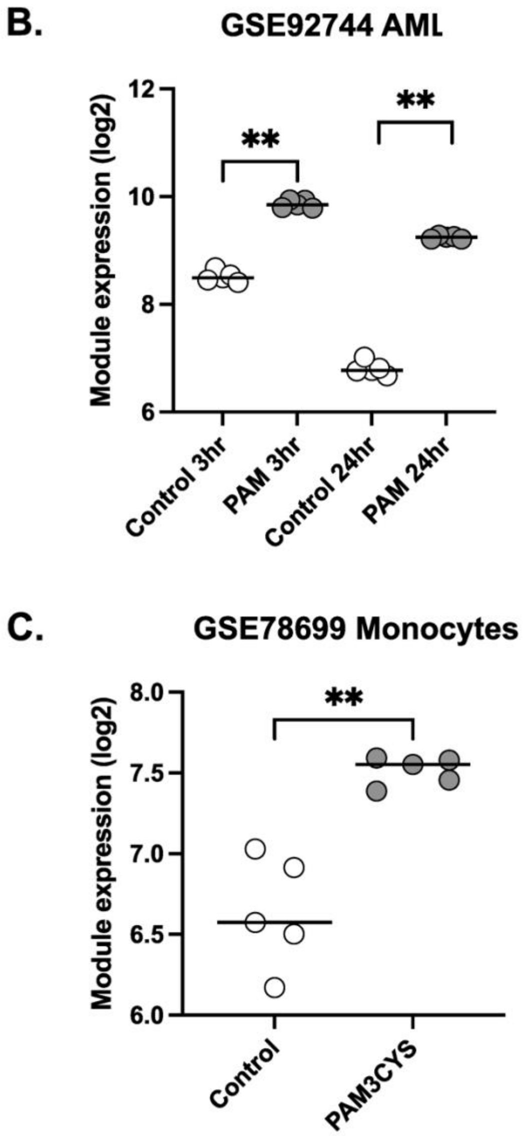

Supplementary Figure 1. TLR2 transcriptional module. Module was derived from genes overexpressed in fibroblasts stimulated with TLR2 agonists Pam_2CSK_4 and/or FSL-1 for 6 hours relative to unstimulated controls, listed in (A). Performance was validated using RNAseq data derived from Acute Myeloid Leukemia cells (B) and monocytes stimulated with Pam_3CSK_4. Statistical testing was performed using Mann-Whitney test. ** p<0.01.

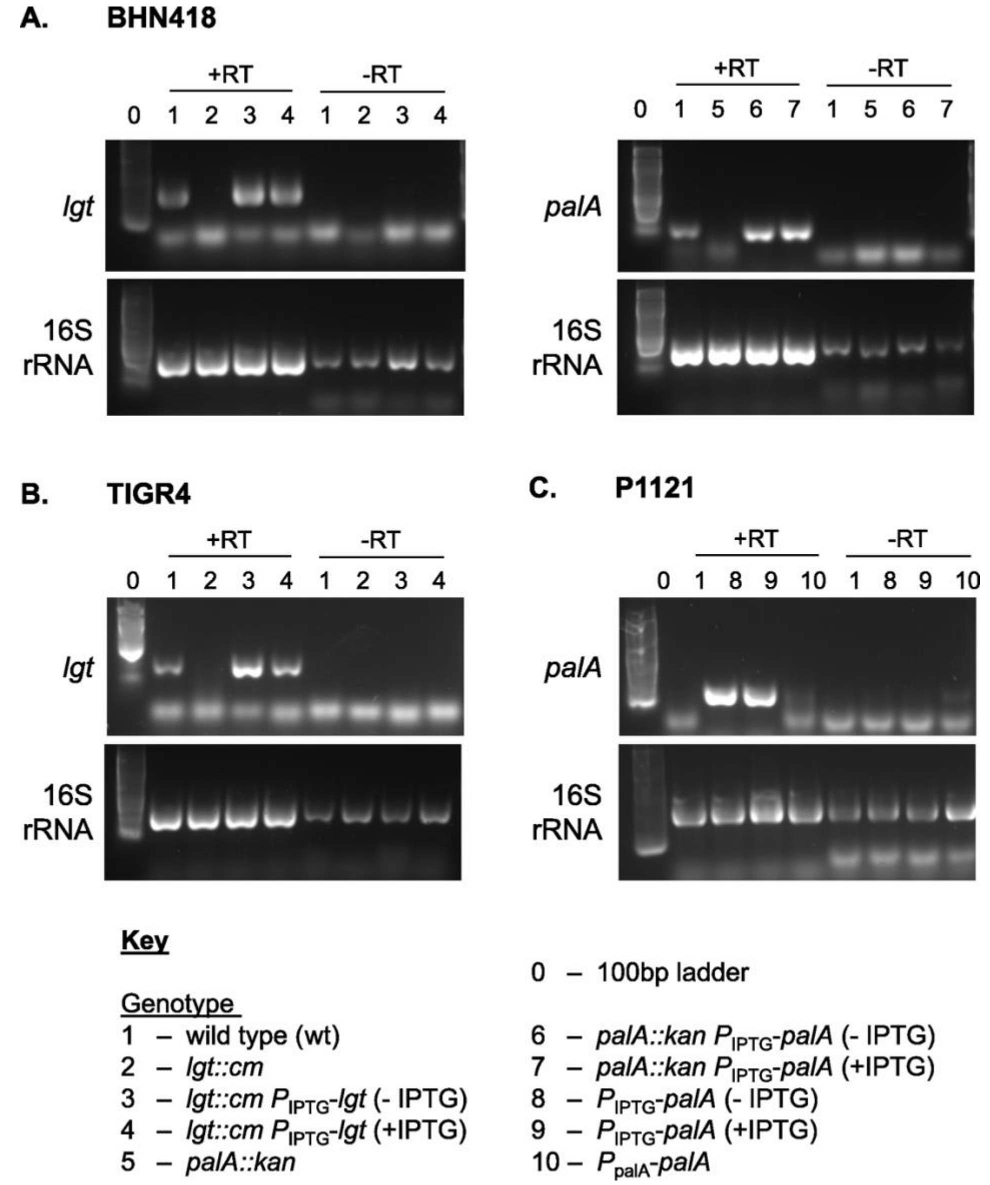

Supplementary Figure 2. Verification of deletion and complementation mutants derived from (A) BHN418, (B) TIGR4 and (C) P1121 via semi-quantitative PCR. DNAse-treated RNA samples were used to generate cDNA (+RT) with a no RT control (-RT) to control for carryover DNA. The generated cDNA were used in a modified qPCR protocol using OneTaq mastermix with primers specific for *lgt*, *palA* and 16S rRNA. Since 16s rRNA would not be eliminated with DNAse treatment, amplification is seen with the -RT samples.

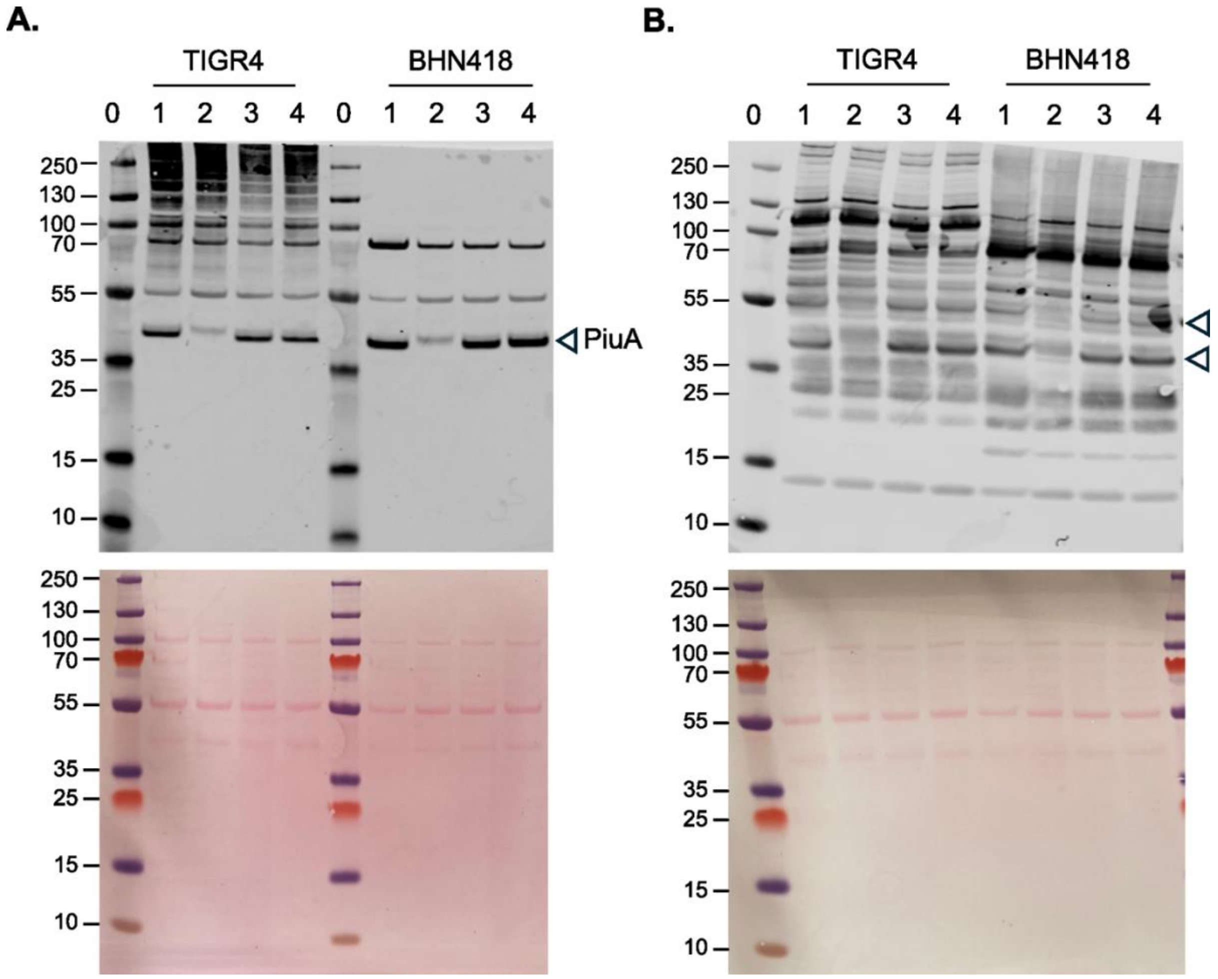

Supplementary Figure 3. Verification of deletion and complementation mutants derived from TIGR4 and BHN418 using immunoblotting. Immunoblotting of whole cell lysates from TIGR4 and BHN418 (1) wild type, (2) *lgt::cm,* (3) *lgt::cm* P_{IPTG}-*lgt* (- IPTG), (4) *lgt::cm* P_{IPTG}-*lgt* (+1mM IPTG) strains was performed with (A) antisera from mice immunized with polysaccharide conjugated to the lipoprotein PiuA and (B) human intravenous immunoglobulin. (Top) Immunoblotting results. Arrows point to PiuA/possible lipoproteins that are lost from the cell surface due to mutation of *lgt*. (Bottom) Membrane stained with Ponceau S to check for equal loading. Approximately 3.5 µg and 2 µg protein lysate were loaded for (A) and (B), respectively. Lane 0 was loaded with PageRuler Plus Prestained Protein Ladder.

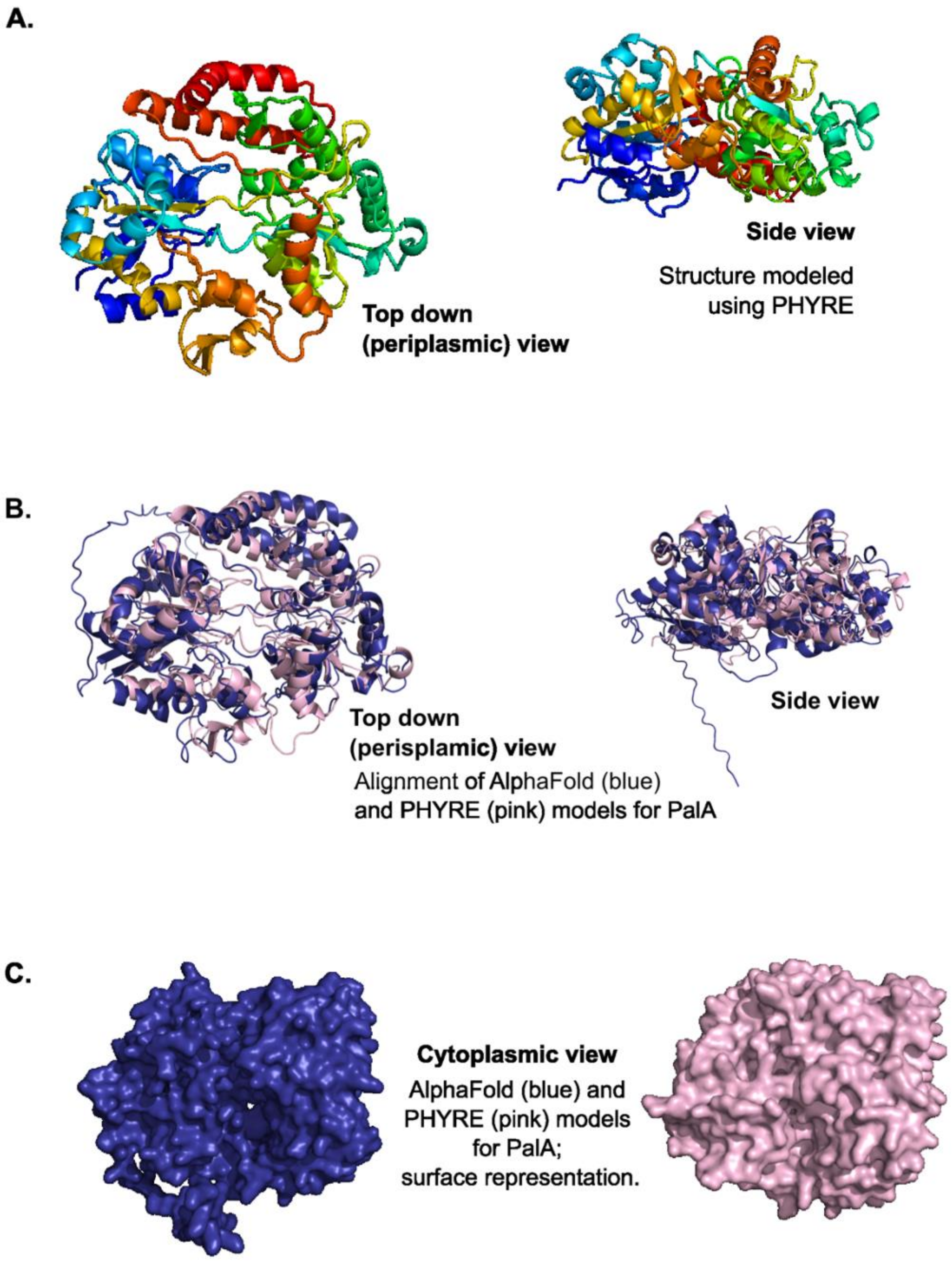

Supplementary Figure 4. Predicted PalA structures using AlphaFold2 and PHYRE algorithms. (A) Predicted PalA structure modeled using PHYRE. (B) Alignment of AlphaFold2 (blue) and PHYRE (pink) models for PalA. (C) Surface representation of AlphaFold2 and PHYRE models for PalA, showing small conformational differences in the accessibility of the potential substrate binding pocket.

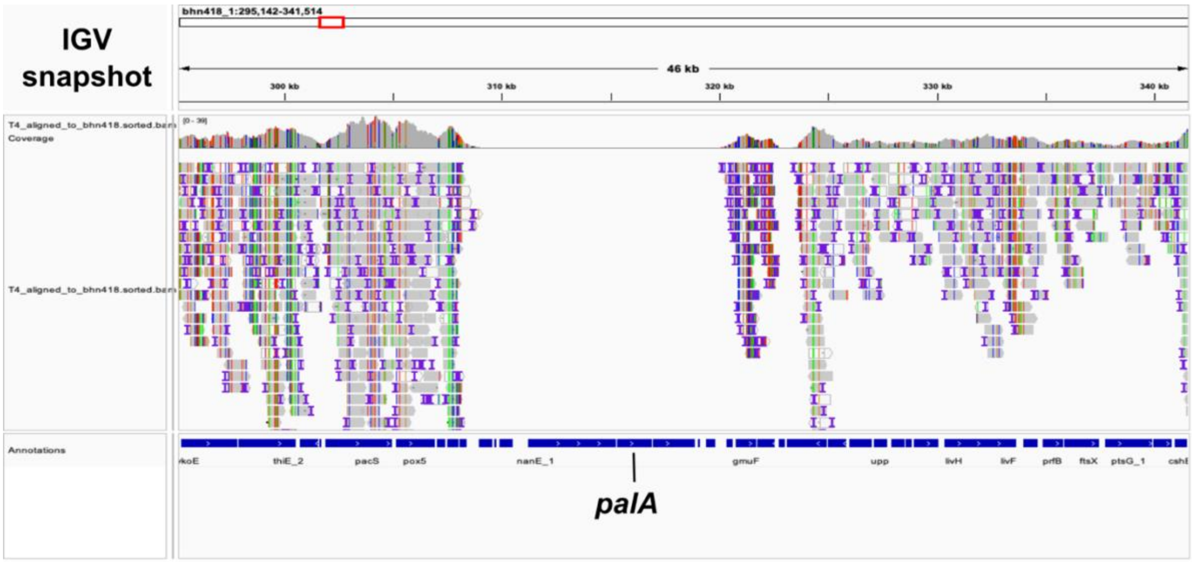

Supplementary Figure 5. IGV snapshot demonstrating lack of TIGR4 sequencing reads mapping to the putative *palA* genetic island.

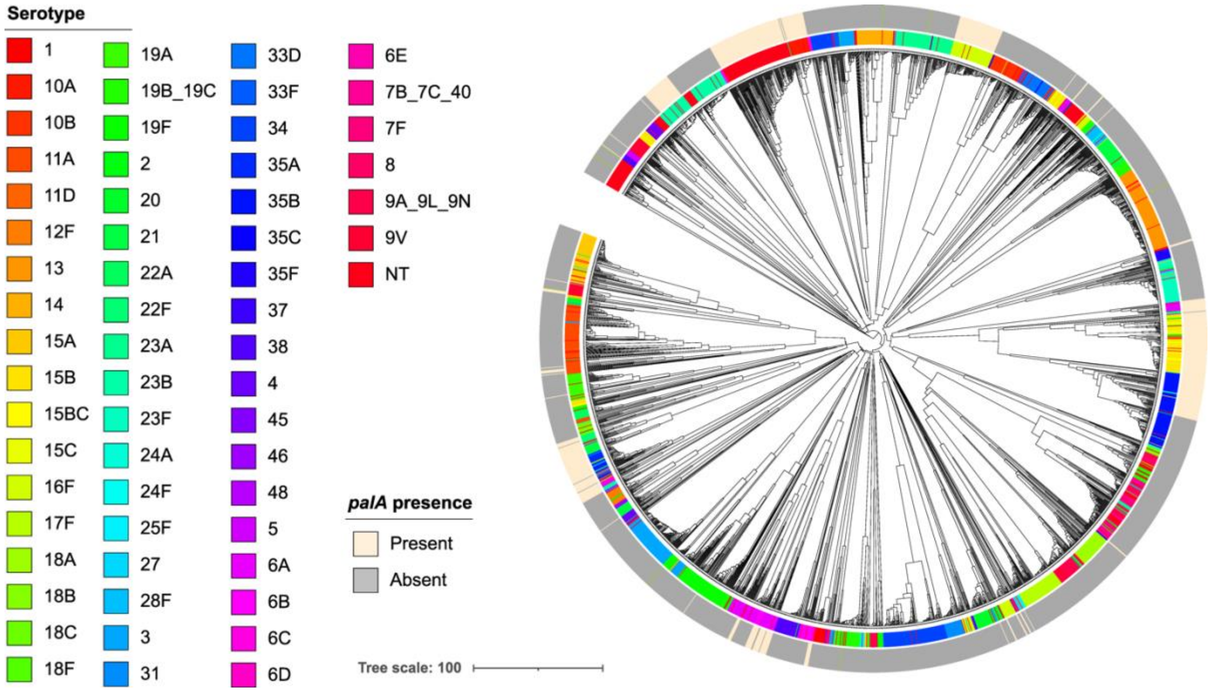

Supplementary Figure 6. *palA* is encoded by diverse pneumococcal strains, with no clear association to specific serotypes. Presence of *palA* mapped onto a hierarchal clustering tree based on metabolic type, constructed using whole genome sequences of 2806 carriage isolates from Malawi (1).

Supplementary Table 1. Summary of TBLASTN results against whole genome sequences of various pneumococcal mutants confirming the presence/absence of target genes *lgt*, *palA*, *cm* and *kan.*

		Gene presence/absence			
**Strain**	**Genotype**	***lgt***	***palA***	***cm***	***kan***
TIGR4	WT Serotype 4	X			
ECSPN100	[TIGR4] *lgt::cm*			X	
ECSPN106	[TIGR4] *lgt::cm* P_{IPTG}-*lgt* (comp)	X		X	
BHN418	WT Serotype 6B	X	X		
ECSPN200	[BHN418] *lgt::cm*		X	X	
ECSPN210	[BHN418] *lgt::cm* P_{IPTG}-*lgt*	X	X	X	
ECSPN211	[BHN418] *palA::kan*	X			X
ECSPN213	[BHN418] *palA::kan* P_{IPTG}-*palA*	X	X		X

Supplementary Table 2. Presence of *palA* in the whole genome sequences of 2806 carriage isolates from Malawi, stratified by serotype.

**Vaccine type (PCV13)**	**Serotype**	***palA* prevalence (%) (*palA* present/total isolates)**
Overall PCVPA dataset		20.21 (567/2806)
PCV13 Vaccine type (VT)	*All VT*	11.11 (77/693)
	**6A**	**41.94 (39/93)**
	**6B**	**38.71 (12/31)**
	9V	14.29 (6/42)
	19F	5.8 (8/138)
	18C	4.76 (1/21)
	3	4.44 (6/135)
	19A	4.35 (2/46)
	23F	4.11 (3/73)
	1	0 (0/13)
	4	0 (0/31)
	5	0 (0/9)
	7F	0 (0/4)
	14	0 (0/57)
Non-vaccine type (NVT)	*All NVT*	20.70 (362/1749)
	**35A**	**100 (28/28)**
	6C	100 (5/5)
	6D	100 (2/2)
	35C	100 (1/1)
	37	100 (1/1)
	**22F**	**85.71 (12/14)**
	**10A**	**85.29 (29/34)**
	**15BC**	**71.43 (15/21)**
	**15B**	**68.42 (52/76)**
	6E	66.67 (2/3)
	**16F**	**63.83 (60/94)**
	**35B**	**52.63 (60/114)**
	**15C**	**48.15 (13/27)**
	**23B**	**43.69 (45/103)**
	**21**	**20.29 (14/69)**
	8	10 (2/20)
	11A	9.43 (10/106)
	15A	6.02 (5/83)
	28F	4.35 (1/23)
	18A	3.77 (2/53)
	20	2.13 (1/47)
	9A_9L_9N	2.08 (1/48)

	34	0.77 (1/130)
	2	0 (0/3)
	7B_7C_40	0 (0/56)
	10B	0 (0/44)
	11D	0 (0/1)
	12F	0 (0/19)
	13	0 (0/123)
	17F	0 (0/66)
	18B	0 (0/2)
	18F	0 (0/1)
	19B_19C	0 (0/68)
	22A	0 (0/15)
	23A	0 (0/78)
	24A	0 (0/6)
	24F	0 (0/3)
	25F	0 (0/5)
	27	0 (0/4)
	31	0 (0/27)
	33D	0 (0/44)
	33F	0 (0/8)
	35F	0 (0/15)
	38	0 (0/47)
	45	0 (0/3)
	46	0 (0/6)
	48	0 (0/3)
Nontypable (NT)	**NT**	**35.16 (128/364)**

Serotypes in **bold** are represented by ≥10 clinical isolates and have above average *palA* carriage rate.

Supplementary Table 3. Primers used in this study. Restriction enzyme sites are underlined.

Primer Designation	Sequence (5'-3')	References
Used in qPCR and semi-quantitative PCR		
gapdh-RT-F	CGGATTTGGTCGTATTGG	This work
gapdh-RT-R	AGATGGTGATGGGATTTC	This work
CXCL10-RT-F	CCTGCTTCAAATATTTCCC	This work
CXCL10-RT-R	CCTTCCTGTATGTGTTTGGA	This work
ifnb-RT-F	CTTGGATTCCTACAAAGAAGC	This work
ifnb-RT-R	CATCTCATAGATGGTCAATGC	This work
IFNL1 RT-PCR F	GCCTCCTCACGCGAGACCTC	(2)
IFNL1 RT-PCR R	GGAGTAGGGCTCAGCGCATA	(2)
IFNL3 RT-PCR F	TGGCCCTGACGCTGAAGGTT	(2)
IFNL3 RT-PCR R	CGTGGGCTGAGGCTGGATAC	(2)
16S rRNA RT F	GGTGAGTAACGCGTAGGTAA	(3)
16S rRNA RT R	ACGATCCGAAAACCTTCTTC	(3)
lgt RT F	GCCTAGCGTTATGATTGCTC	This work
lgt RT R	CGAAAGCCGAAGAACATGAG	This work
palA-RT-F	TCAACGTAATGGCTATGAAGGCT	This work
palA-RT-R	CCCAGTCAGGGTGAAGTACG	This work
Used to generate transforming DNA for making ECSPN100, ECSPN200.		
Sp1411F	GAGTCATCAAGAGCTTCGG	(4)
Cm-1411R	GCCTAATGACTGGCTTTTATAAATGTTAGAAGTTGCATATATTC	(4)
Cm-1413F	ACATTATCCATTAAAAATCAAATCAAGCATTTTGCACCTCATTT	(4)
Sp1413R	CATGCCTTCCAACAGCCG	(4)
lgt-CmF	TTATAAAAGCCAGTCATTAG	(4)
lgt-CmR	TTTGATTTTTAATGGATAATG	(4)
For cloning pEC210, pEC211.		
BamHI lgt comp F	GATAGGATCCACGAACGACTGACAAG	This work
XhoI lgt comp R	GATACTCGAGACATTTAGTTTTCCTCCTCTG	This work
Used to generate transforming DNA for making ECSPN211.		
00322 up F	GCTTCAATGTACCACGAAG	This work
kan-00322 up R	ACGAACTCCAATTCACTGTTCAGCTTTACCATAATAAGACCTC	This work
kan-00322 down F	TCTGAAGTACATCCGCAACTAGACTAAATGGTAGCTCTCTG	This work
00322 down R	CATCATTCATAAAATGGTCGTC	This work
pABG kanF	GAACAGTGAATTGGAGTTCG	This work

pABG kanR	AGTTGCGGATGTACTTCAG	This work
For cloning pEC213.		
BamHI 00322 comp F2	AGGTAGGATCCGTGGAAATGGTAATCACACTG	This work
XhoI 0322 comp R	GGTTATCTCGAGGGCAGTAGTAGCTCTCTG	This work

REFERENCES

1. Obolski U, Swarthout TD, Kalizang'oma A, Mwalukomo TS, Chan JM, Weight CM, Brown C, Cave R, Cornick J, Kamng'ona AW, Msefula J, Ercoli G, Brown JS, Lourenço J, Maiden MC, French N, Gupta S, Heyderman RS. 2023. The metabolic, virulence and antimicrobial resistance profiles of colonising *Streptococcus pneumoniae* shift after PCV13 introduction in urban Malawi. Nat Commun 14:7477.
2. Ramos I, Smith G, Ruf-Zamojski F, Martínez-Romero C, Fribourg M, Carbajal EA, Hartmann BM, Nair VD, Marjanovic N, Monteagudo PL, DeJesus VA, Mutetwa T, Zamojski M, Tan GS, Jayaprakash C, Zaslavsky E, Albrecht RA, Sealfon SC, García-Sastre A, Fernandez-Sesma A. 2019. Innate immune response to influenza virus at single-cell resolution in human epithelial cells revealed paracrine induction of interferon lambda 1. J Virol 93:10.1128/jvi.00559-19.
3. Ogunniyi AD, Giammarinaro P, Paton JC. 2002. The genes encoding virulence-associated proteins and the capsule of *Streptococcus pneumoniae* are upregulated and differentially expressed *in vivo*. Microbiology 148:2045–2053.
4. Chimalapati S, Cohen JM, Camberlein E, MacDonald N, Durmort C, Vernet T, Hermans PWM, Mitchell T, Brown JS. 2012. Effects of deletion of the *Streptococcus pneumoniae* lipoprotein diacylglyceryl transferase gene *lgt* on ABC transporter function and on growth *in vivo*. PLoS One 7:e41393.
